# Impaired 1,25 dihydroxyvitamin D_3_ action and hypophosphatemia underlie the altered lacuno-canalicular remodeling observed in the Hyp mouse model of XLH

**DOI:** 10.1371/journal.pone.0252348

**Published:** 2021-05-27

**Authors:** Ye Yuan, Supriya Jagga, Janaina S. Martins, Rakshya Rana, Paola Divieti Pajevic, Eva S. Liu

**Affiliations:** 1 Harvard Medical School, Boston, Massachusetts, United States of America; 2 Division of Endocrinology, Diabetes, Hypertension, Brigham and Women’s Hospital, Boston, Massachusetts, United States of America; 3 Endocrine Unit, Massachusetts General Hospital, Boston, Massachusetts, United States of America; 4 Department of Translational Dental Medicine, Boston University School of Dental Medicine, Boston, Massachusetts, United States of America; VA Loma Linda Healthcare System, UNITED STATES

## Abstract

Osteocytes remodel the perilacunar matrix and canaliculi. X-linked hypophosphatemia (XLH) is characterized by elevated serum levels of fibroblast growth factor 23 (FGF23), leading to decreased 1,25 dihydroxyvitamin D_3_ (1,25D) production and hypophosphatemia. Bones from mice with XLH (Hyp) have enlarged osteocyte lacunae, enhanced osteocyte expression of genes of bone remodeling, and impaired canalicular structure. The altered lacuno-canalicular (LCN) phenotype is improved with 1,25D or anti-FGF23 antibody treatment, pointing to roles for 1,25D and/or phosphate in regulating this process. To address whether impaired 1,25D action results in LCN alterations, the LCN phenotype was characterized in mice lacking the vitamin D receptor (VDR) in osteocytes (VDR^f/f;DMP1Cre+^). Mice lacking the sodium phosphate transporter NPT2a (NPT2aKO) have hypophosphatemia and high serum 1,25D levels, therefore the LCN phenotype was characterized in these mice to determine if increased 1,25D compensates for hypophosphatemia in regulating LCN remodeling. Unlike Hyp mice, neither VDR^f/f;DMP1Cre+^ nor NPT2aKO mice have dramatic alterations in cortical microarchitecture, allowing for dissecting 1,25D and phosphate specific effects on LCN remodeling in tibial cortices. Histomorphometric analyses demonstrate that, like Hyp mice, tibiae and calvariae in VDR^f/f;DMP1Cre+^ and NPT2aKO mice have enlarged osteocyte lacunae (tibiae: 0.15±0.02μm^2^(VDR^f/f;DMP1Cre-^) vs 0.19±0.02μm^2^(VDR^f/f;DMP1Cre+^), 0.12±0.02μm^2^(WT) vs 0.18±0.0μm^2^(NPT2aKO), calvariae: 0.09±0.02μm^2^(VDR^f/f;DMP1Cre-^) vs 0.11±0.02μm^2^(VDR^f/f;DMP1Cre+^), 0.08±0.02μm^2^(WT) vs 0.13±0.02μm^2^(NPT2aKO), p<0.05 all comparisons) and increased immunoreactivity of bone resorption marker Cathepsin K (Ctsk). The osteocyte enriched RNA isolated from tibiae in VDR^f/f;DMP1Cre+^ and NPT2aKO mice have enhanced expression of matrix resorption genes that are classically expressed by osteoclasts (*Ctsk*, *Acp5*, *Atp6v0d2*, *Nhedc2*). Treatment of Ocy454 osteocytes with 1,25D or phosphate inhibits the expression of these genes. Like Hyp mice, VDR^f/f;DMP1Cre+^ and NPT2aKO mice have impaired canalicular organization in tibia and calvaria. These studies demonstrate that hypophosphatemia and osteocyte-specific 1,25D actions regulate LCN remodeling. Impaired 1,25D action and low phosphate levels contribute to the abnormal LCN phenotype observed in XLH.

## Introduction

Osteocytes are terminally differentiated osteoblast cells that act as mechanosensors for the skeleton and play an important role in the regulation of phosphate homeostasis [1–3]. These cells reside in small cavities in the bone called lacunae, surrounded by mineralized matrix [4]. The canaliculi are a system of channels that extend from the osteocytes through which nutrients and small molecules are transported between cells [3–5]. The lacunae, together with the canaliculi, form the lacuno-canalicular network (LCN). During lactation in mice, the enlarged osteocyte lacunae are associated with an enhanced expression of genes classically expressed by osteoclasts to regulate bone resorption, such as cathepsin K (*Ctsk*) and tartrate-resistant acid phosphatase (*Acp5*) [4, 6, 7]. Deletion of parathyroid hormone receptor 1 (PTHR1) in lactating mice was shown to prevent enhanced perilacunar remodeling [6]. Moreover, administration of PTH related peptide (PTHrP) to rats also resulted in enlarged osteocyte lacunae [8]. These studies suggest that PTH/PTHrP action enhances perilacunar matrix resorption to enable calcium release.

X-linked hypophosphatemia (XLH) is the most common form of inherited rickets caused by a mutation in the phosphate regulating gene with homology to endopeptidase located on the X chromosome (PHEX), which leads to elevated serum levels of fibroblast growth factor 23 (FGF23) [9–11]. High serum levels of FGF23 downregulate the renal sodium-phosphate transporters, NPT2a and NPT2c, resulting in increased urinary phosphate wasting [12]. FGF23 also inhibits vitamin D 1-α-hydroxylase, leading to decreased synthesis of 1,25 dihydroxyvitamin D_3_ (1,25D) [13, 14]. Both calvaria and tibiae of mice with XLH (Hyp) have enlarged lacunae and impaired canalicular organization [15]. Similar to lactating mice, the enlarged lacunae in Hyp mice are associated with enhanced expression of bone/matrix resorption genes. Administration of daily 1,25D or an antibody targeting FGF23 (FGF23Ab), which also enhances 1,25D action, to Hyp mice restored lacunar size and improved canalicular morphology [15]. The osteocyte expression of genes that regulate perilacunar remodeling were normalized with 1,25D treatment and improved with FGF23Ab treatment. No changes in collagen organization were observed in the cortical bone of control and treated Hyp mice, therefore this parameter could not account for the alterations in canalicular structure [15]. The improvement in the lacuno-canalicular organization with 1,25D and FGF23Ab treatments points to roles for 1,25D and/or phosphate in regulating perilacunar and canalicular remodeling [15].

To address the hypothesis that 1,25D acts directly on osteocytes to modulate osteocyte perilacunar remodeling and canalicular organization, we analyzed the LCN phenotype in mice lacking the vitamin D receptor (VDR) in osteocytes (VDR^f/f;DMP1Cre+^) because these mice maintain normal serum calcium, phosphate, and skeletal microarchitecture [16]. Mice null for NPT2a (NPT2aKO) have hypophosphatemia, which leads to high serum 1,25D levels [17]. Analysis of the LCN phenotype in NPT2aKO mice will determine whether a physiological increase in 1,25D can compensate for low serum phosphate in regulating osteocyte lacunar and canalicular organization. Since Hyp mice have an abnormal cortical LCN phenotype [15] accompanied by significantly impaired cortical microarchitecture [18], the cortical microarchitecture in VDR^f/f;DMP1Cre+^ and NPT2aKO mice was analyzed to determine if changes in the LCN phenotype in these mice was also associated with alterations in cortical bone. Evaluation of the LCN phenotypes of VDR^f/f;DMP1Cre+^ and NPT2aKO mice will demonstrate whether impaired 1,25D action and/or low serum phosphate are physiologic stimuli for enhancing osteocyte perilacunar and canalicular remodeling.

## Materials and methods

### Ethics statement

Animal studies were approved by the institutional animal care committee at Brigham and Women’s Hospital, Boston, MA (Protocol number 2018N000044). This study was performed in accordance with the recommendations in the Guide for Care and Use of Laboratory Animals of the National Institutes of Health. At the time of sacrifice, mice were exposed briefly to isoflurane anesthesia, bled by cheek bleed to exsanguination, and then subjected to cervical dislocation.

### Animal studies

All mice were on a C57BL/6J background, maintained in a virus and parasite free barrier facility and exposed to a 12-hour light/dark cycle. All mice were weaned day 18 onto house chow (0.8% calcium, 0.6% phosphate, PicoLab 5053) and housed in up to 5 mice per cage. Studies were performed in male and female mice null for NPT2a (NPT2aKO) [19] and mice lacking the vitamin D receptor in osteocytes (VDR^f/f;DMP1Cre+^). To ablate *NPT2a* in mice, Beck et al. replaced exons 6 through 10 of the *NPT2a* gene with a neomycin selection cassette via homologous recombination [19]. Mice with osteocyte-specific ablation of VDR were generated by mating mice expressing Cre recombinase under the control of the 9.6kb-Dentin matrix protein 1 (DMP1) promoter (DMP1Cre) [20] with mice bearing exon 4 floxed VDR alleles [16]. The phenotype of male and female mice homozygous for osteocyte specific VDR ablation (VDR^f/f;DMP1Cre+^) was compared to that of age and sex-matched Cre negative littermates homozygous for the VDR floxed allele (VDR^f/f;DMP1Cre-^). Age and sex-matched WT mice, including litter mates of both Hyp and NPT2aKO mice, served as controls. Since the LCN phenotype of Hyp mice was previously characterized [15], age and sex-matched Hyp mice served as additional controls. Equal numbers of male and female mice were included for each genotype for each parameter analyzed. At the time of sacrifice, the weights (g) and body length (mm) of each mouse was measured.

### Serum parameters

All mice were fasted for 2 hours in the morning, after which serum from mice was obtained by cheek bleed. This procedure was performed under isoflurane anesthesia to minimize suffering. Serum calcium and phosphate were measured colorimetrically using kits from Stanbio (Boerne, TX) and Abcam (Cambridge, MA), respectively. Serum PTH and FGF23 were analyzed using the Mouse Intact PTH 1–84 Kit and Mouse Intact FGF23 ELISA Kit, respectively (Immutopics, San Clemente, CA). Serum 1,25D was immunopurified and measured by competitive ELISA (Immunodiagnostic Systems, Gaithersburg, MD).

### Micro-computed tomography (microCT)

A high-resolution desktop micro-tomographic imaging system (microCT40, Scanco Medical AG, Brüttisellen, Switzerland) was used to assess cortical and trabecular bone microarchitecture in the mid-diaphysis in unfixed non-decalcified femurs that were stored in minus 20 degrees in saline soaked gauze. Scans were acquired using a 6 μm^3^ isotropic voxel size, 70 kVP, 114 mAs, 200 ms integration time. Image acquisition and analysis protocols adhered to guidelines for microCT analysis of rodent bones [21].

Cortical bone architecture and mineral density was analyzed in a 300 μm long region (50 transverse slices) at the femoral mid-diaphysis. Analysis was performed on manually contoured regions that included the whole bone cross-section as well as a region that just included the cortex. Images were subjected to Gaussian filtration and segmented using a fixed threshold of 700 mgHA/cm^3^ and analyzed with the Scanco mid-shaft evaluation script to measure the following cortical parameters: total cross-sectional area (Tt.Ar, mm^2^), medullary area (Ma.Ar, mm^2^) cortical bone area (Ct.Ar, mm^2^), cortical bone area fraction (Ct.Ar/Tt.Ar, %), cortical thickness (Ct.Th, mm), cortical porosity (%), and the polar moments of inertia (mm^4^).

Trabecular bone in the distal femoral metaphysis was analyzed in a region beginning 200 μm superior to the growth plate and extending proximally 900 μm (150 transverse slices). The trabecular bone in this region was selected by semi-manually contouring the endocortical region of the bone. Bone was segmented from soft-tissue using a segmentation threshold of 340 mgHA/cm^3^ and analyzed using the standard Scanco trabecular bone morphometry script to measure the following parameters: trabecular bone volume fraction (Tb.BV/TV, %), trabecular thickness (Tb.Th, mm), trabecular number (Tb.N, mm^-1^), and trabecular separation (Tb.Sp, mm).

### Histology

Tibiae and calvariae were fixed in 10% formalin for 24 hours, decalcified in 20% EDTA/PBS for 2 weeks, and processed for paraffin or frozen sectioning. For frozen sections, decalcified calvariae were subject to sucrose equilibration and then embedded in OCT. For silver staining, paraffin sections of tibiae were decalcified in 20% EDTA (pH 8) for 15 minutes and then incubated in silver nitrate solution (2/3 volume of 50% silver nitrate with 1/3 volume of 2% gelatin and 1% formic acid) at 37°C in the dark for 50 minutes. Following a rinse in water, sections were immersed in 15% sodium thiosulfate overnight. To visualize the calvarial canalicular network, frozen decalcified calvarial sections were fixed in 4% paraformaldehyde in PBS and permeabilized with 0.5% Saponin in PBS. After blocking sections in 5% BSA in PBS, calvariae were incubated with 1:300 phalloidin conjugated to iFluor-488 (Abcam, Cambridge, MA, ab176753), followed by DAPI to stain for nuclei. The calvarial canaliculi were then imaged using confocal microscopy, with Z-stack images (20 micron thickness [22] at 0.75 micron plane separation) being obtained with a Zeiss LSM 800 with Airyscan system on a Zeiss Axio Observer Z1 inverted microscope (Zeiss, Oberkochen, Germany) using a 63x oil objective and ZEN Blue 2.6 software (Zeiss).

To analyze the tibial and calvarial canalicular structure, the number of dendritic processes extending from each lacunae were manually quantitated as previously described [15]. Briefly, for each mouse, 10 osteocytes from 3 regions of the cortical bone were visualized at 40x magnification. The regions of cortical bone analyzed were located at the tibial mid-shaft contralateral to the tibia-fibula junction midway between the periosteal and endosteal surfaces. To analyze calvarial canalicular structure, three-dimensional images using Z-stack images obtained from the confocal microscope were rendered using ZEN Blue 2.6 (Zeiss, Oberkochen, Germany). The number of dendritic processes per osteocyte on 10 individual osteocytes from the left and right parietal bones, midway between the sagittal and temporal sutures, were quantified. The investigators performing these analyses were blinded to the identity of each sample.

### Histomorphometry

Periosteocytic lacunar area (Lac.Ar) in the lateral tibial cortex, 5 mm above the tibia-fibula junction [15], as well as the Lac.Ar in the left and right parietal bones of the calvaria, midway between the sagittal and temporal sutures and midway between the coronal and lambdoid sutures, was quantitated on paraffin sections at a magnification of 40× using an Osteomeasure image analyzer (Osteometrics, Atlanta, GA, USA). As previously reported, because Hyp cortices have decreased osteocyte number relative to WT [15], Lac.Ar was normalized to osteocyte number. The investigator performing these analyses was blinded to the identity of each sample.

### Osteocyte gene expression

Osteocyte enriched RNA was isolated from mouse tibia as previously described [15]. Tibiae were isolated from day 30 mice, after which the epiphyses were cut off and the diaphysis subjected to centrifugation and flushing with PBS to remove the bone marrow. The remaining tibial cortices were then subjected to sequential digestions in 0.2% type 1 collagenase (Worthington, Lakewood, NJ)/0.05% trypsin (Thermo Fisher Scientific, Waltham, MA) in α-MEM media/25 mM Hepes/0.1% BSA or 0.53 mM EDTA/0.05% trypsin in PBS/0.1% BSA. Bone explants were shaken for 30 minutes at 37°C in a ThermoMixer R (Eppendorf, Hauppauge, NY). Digested tibial cortices were homogenized in Trizol (Thermo Fisher Scientific). Total RNA was precipitated using 100% alcohol, purified using the *Quick-*RNA Tissue/Insect Kit (Zymo Research, Irvine, CA), and reverse transcribed with SuperScript^TM^ II (Roche). Quantitative real time-PCR was performed using the QuantiTect SYBR Green RT-PCR kit (Qiagen, Germantown, MD) on Light Cycler® 480 II (Roche, San Francisco, CA). Gene expression was quantitated relative to beta-actin and then normalized to that of WT for each sample, using the methods of Livak and Schmittgen [23]. Hyp and NPT2aKO osteocyte gene expression was normalized to that of WT litter mates. VDR^f/f;DMP1Cre-^ and VDR^f/f;DMP1Cre+^ RNA was isolated together with either a WT/Hyp or WT/NPT2aKO litter, therefore VDR^f/f;DMP1Cre-^ and VDR^f/f;DMP1Cre+^ osteocyte gene expression was normalized to that of WT litter mates of Hyp and NPT2aKO mice isolated concurrently.

### Immunohistochemistry

Immunohistochemistry for cathepsin K was performed as previously described [15]. Paraffin sections were blocked with 10% heat inactivated fetal bovine serum and then incubated with anti-cathepsin K antibody (rabbit polyclonal antibody, 1:200; Abcam, Cambridge, MA; ab19027). Signal detection was performed using biotinylated goat-anti rabbit secondary antibody (Vector, Burlingame, CA; BA-1000) followed by incubation with avidin-biotin complex (Vecta Stain; PK-6100).

### Cell culture

The osteocyte cell line Ocy454 [24] was maintained in α-MEM supplemented with 10% fetal bovine serum (Gibco, Waltham, MA) with 1% antibiotic/anti-mycotic (Gibco). Cells were plated at 2x10^4^ cells, allowed to grow to confluency in 33°C, and then transferred to 37°C for 7 days. Cells were then starved in 2% FBS overnight. For the 1,25D treatment protocol, starved cells were treated with vehicle or 10^-8^M 1,25D for 24 hours. For the phosphate treatment protocol, starved cells were incubated with 7mM sodium sulfate (control) or 7 mM sodium phosphate [25] for 8 hours. Total RNA was then isolated using the PureLink RNA Kit (Invitrogen, Carlsbad, CA) and subjected to reverse transcription followed by RT-qPCR as described above. Cells incubated at 37°C were routinely assayed for sclerostin (SOST) expression to assure maintenance of their osteocytic phenotype.

### Statistical analysis

All data shown are reported as mean ± standard deviation (SD). One-way ANOVA followed by Fisher’s least significant difference (LSD) test was used to analyze significance between all control and treatment groups [15, 18]. Significance was defined as a P< 0.05. Since male and female mice have uniform phenotypes within each genotype of mice, analyses are performed with each group including mice of both sexes.

## Results

### Generation of mice lacking the Vdr in osteocytes

**Fig 1:**
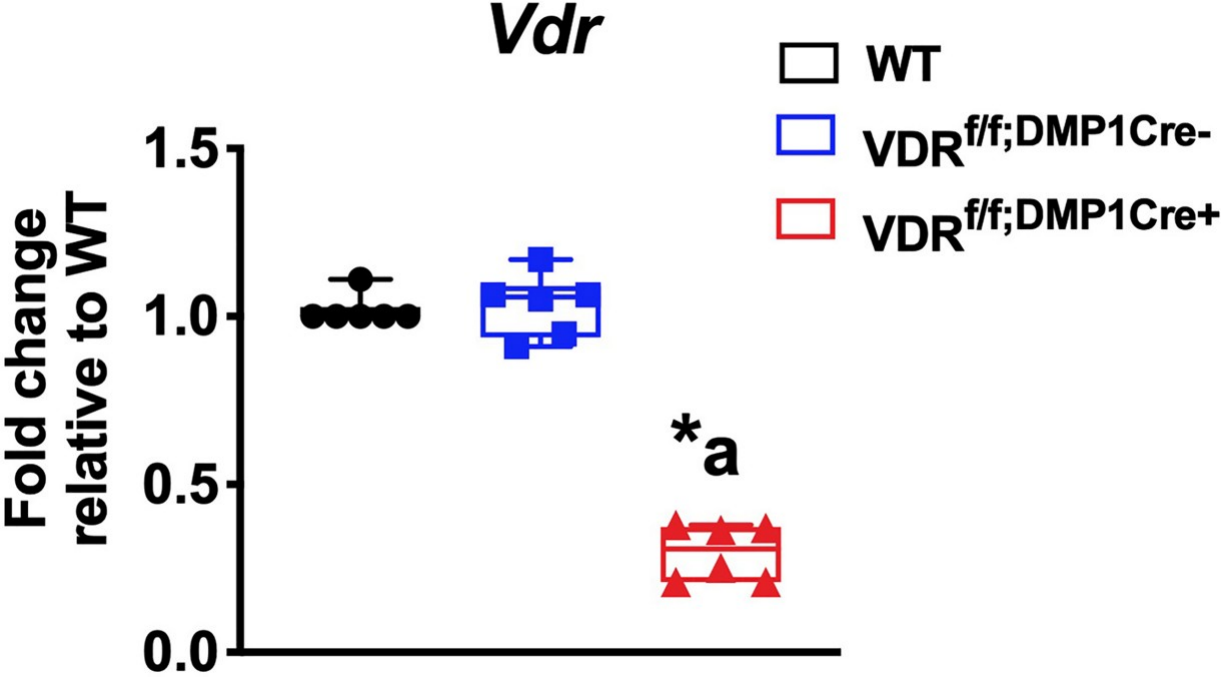
Generation of the VDR^f/f;DMP1Cre+^ mice. mRNA expression of *Vdr* is quantitated in osteocyte-enriched tibiae from d30 WT, VDR^f/f;DMP1Cre-^, and VDR^f/f;DMP1Cre+^ mice. Data normalized to actin and represented as fold change relative to WT. Data are representative of that obtained from 6 mice per genotype, with each group including 3 mice of either sex. * = p<0.05 versus WT, a = p<0.05 versus VDR^f/f;DMP1Cre-^.

**Fig 2:**
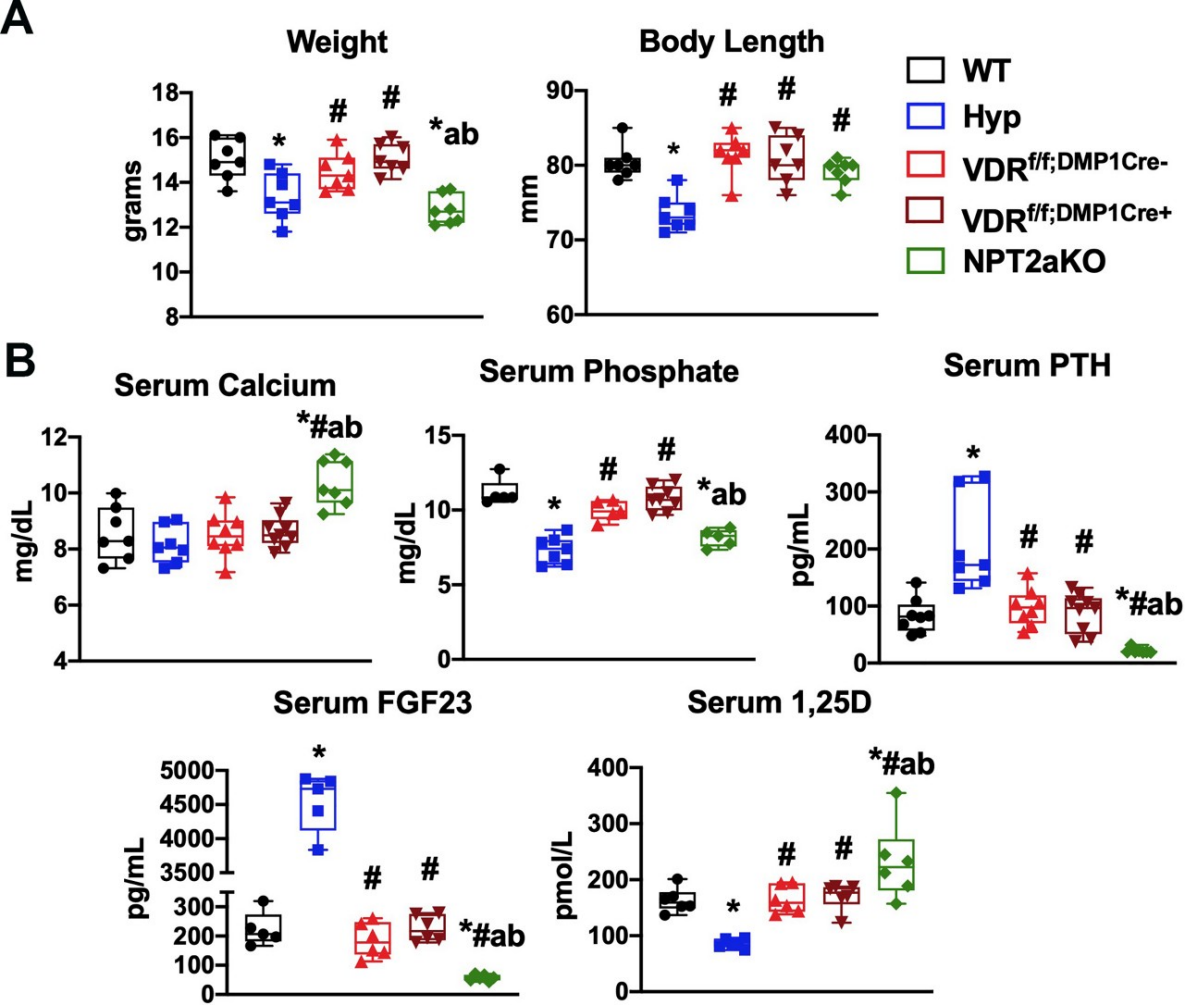
Body weight and length and serum parameters for VDR^f/f;DMP1Cre+^, NPT2aKO, and control mice. (A) Body weight and length and (B) serum mineral ions and hormones were measured for d30 WT, Hyp, VDR^f/f;DMP1Cre-^, VDR^f/f;DMP1Cre+,^ and NPT2aKO mice. For weight, body length, and serum calcium and PTH, data are representative of that obtained from at 7–9 mice per genotype, with each group including 3–5 mice of either sex. For serum phosphate, FGF23, and 1,25D, data are representative of at least 5 mice per genotype, with each group including 2–4 mice of either sex. * p<0.05 vs WT, # p<0.05 vs Hyp, a p<0.05 vs VDR^f/f;DMP1Cre-^, b p<0.05 vs VDR^f/f;DMP1Cre+^.

Hyp cortices have enlarged osteocyte lacunar area, which is normalized by enhanced 1,25D action achieved by treatment with either 1,25D or FGF23Ab [15]. To determine if 1,25D acts directly on osteocytes to regulate LCN remodeling, mice with an osteocyte specific deletion of the *Vdr* (VDR^f/f;DMP1Cre+^) were generated by mating mice expressing the Cre-recombinase under the control of the DMP1 promoter (9.6kb-DMP1Cre) [20] to mice with exon 4 floxed *Vdr* alleles (VDR^f/f^) [16]. To confirm deletion of the *Vdr* in osteocytes, mRNA expression for *Vdr* was quantitated in osteocyte enriched tibiae from VDR^f/f;DMP1Cre+^ and control (WT and VDR^f/f;DMP1Cre-^) mice. Expression of exon 4-containing *Vdr* transcripts in VDR^f/f;DMP1Cre+^ osteocyte enriched tibiae is reduced by 71.9±9.5% compared to VDR^f/f;DMP1Cre-^ tibiae, demonstrating efficient *Vdr* deletion. (Fig 1). Expression of *Vdr* is not different between WT and VDR^f/f;DMP1Cre-^ tibiae (Fig 1). VDR^f/f;DMP1Cre+^ mice were born at a normal Mendelian frequency and had similar weights and body lengths to VDR^f/f;DMP1Cre-^ control mice (Fig 2A). They also have normal serum calcium, phosphate, PTH, FGF23, and 1,25D levels (Fig 2B).

The improvement in the LCN phenotype in Hyp bones with either 1,25D or FGF23Ab therapy also supports a role for phosphate in regulating LCN remodeling [15]. NPT2aKO mice have hypophosphatemia, leading to a physiologic elevation in 1,25D levels [17] and decreased serum FGF23 levels (Fig 2B). The high 1,25D levels, which are 1.4±0.4 fold higher than WT (Fig 2B), result in hypercalcemia and suppressed serum PTH levels [17], as confirmed in Fig 2B. NPT2aKO mice have decreased body weight and normal body length (Fig 2A). Evaluation of the LCN phenotype in NPT2aKO mice was performed to determine if high 1,25D levels compensate for hypophosphatemia or if low serum phosphate levels are critical for the modulation of LCN remodeling.

### VDR^f/f;DMP1Cre+^ and NPT2aKO mice do not have significantly altered cortical microarchitecture

**Fig 3:**
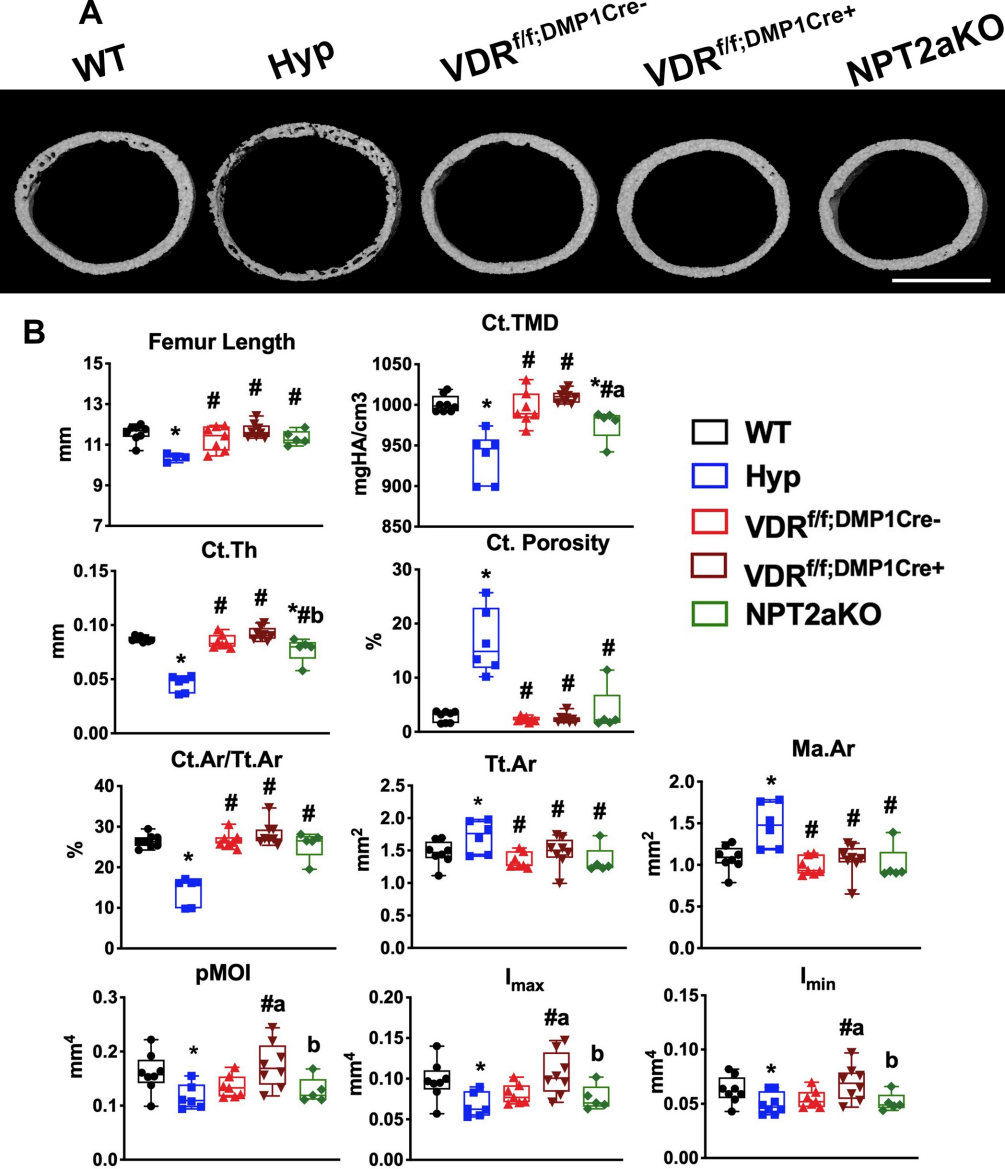
VDR^f/f;DMP1Cre+^ and NPT2aKO mice do not have significantly altered cortical microarchitecture. MicroCT was performed on d30 femurs isolated from WT, Hyp, VDR^f/f;DMP1Cre-^, VDR^f/f;DMP1Cre+^ and NPT2aKO mice. (A) Representative images of microCT scans are shown. Scale bar = 0.5 mm. (B) Cortical microCT parameters (Femur length, cortical tissue mineral density (Ct.TMD), cortical thickness (Ct.Th), cortical porosity (Ct.porosity), cortical area fraction (Ct.Ar/Tt.Ar), total area (Tt.Ar), medullary area (Ma.Ar), and polar moments of inertia (pMOI, I_max_, I_min_)) are shown. Data are representative of that obtained from at least 5 mice per genotype, with NPT2aKO mice including 2–3 mice of either sex and all other groups including 3–4 mice of either sex. * p<0.05 vs WT, # p<0.05 vs Hyp, a p<0.05 vs VDR^f/f;DMP1Cre-^, b p<0.05 vs VDR^f/f;DMP1Cre+^.

Hyp mice have significantly impaired cortical microarchitecture, including decreased cortical thickness and cortical area fraction [18], suggesting the alterations in the cortical bone could contribute to the abnormal osteocyte LCN phenotype observed in the cortices of these mice [18]. To determine whether VDR^f/f;DMP1Cre+^ or NPT2aKO mice have abnormal cortical microarchitecture, microCT analyses were performed in VDR^f/f;DMP1Cre+^ and NPT2aKO bones (Fig 3A and 3B). Neither VDR^f/f;DMP1Cre+^ nor NPT2aKO have bone deformities, as confirmed by representative microCT images of the mid to distal femurs (S1 Appendix). While Hyp femurs are shorter than WT, both VDR^f/f;DMP1Cre+^ and NPT2aKO femurs are normal in length (Fig 3B). VDR^f/f;DMP1Cre-^ control femurs do not have altered cortical microarchitecture relative to WT (Fig 3A and 3B). Compared to WT, Hyp femurs have significantly decreased tissue mineral density (TMD), cortical thickness (Ct.Th), and cortical area fraction (Ct.Ar/Tt.Ar) and increased cortical porosity (Fig 3B). Both VDR^f/f;DMP1Cre+^ and NPT2aKO femurs have normal cortical porosity and cortical area fraction. Although Hyp femurs have increased total cross sectional area (Tt.Ar) and medullary area (Ma.Ar), these parameters are normal in VDR^f/f;DMP1Cre+^ and NPT2aKO femurs. While deletion of *Vdr* in osteocytes does not alter TMD or Ct.Th, NPT2aKO femurs have slight but significant decreases in TMD and Ct.Th compared to WT. MicroCT evaluation of inferred biomechanical parameters demonstrated a significant decrease in polar moments of inertia (pMOI, I_max_, and I_min_) in Hyp femurs, but did not reveal any differences in these parameters in VDR^f/f;DMP1Cre+^ or NPT2aKO femurs relative to WT and VDR^f/f;DMP1Cre-^ controls (Fig 3B). These results show that VDR^f/f;DMP1Cre+^ and NPT2aKO mice do not have significantly impaired cortical microarchitecture, therefore alterations in cortical parameters are likely do not contribute to alterations in the LCN phenotype characterized in these mice.

Although the LCN phenotype is analyzed in cortical bone, the trabecular microarchitecture parameters of VDR^f/f;DMP1Cre+^ and NPT2aKO femurs and corresponding controls were also quantitated. VDR^f/f;DMP1Cre-^ and VDR^f/f;DMP1Cre+^ mice have normal trabecular microarchitecture (S2 Appendix). In contrast, the changes in trabecular microarchitecture for mice null for NPT2a are similar to that observed in Hyp mice (S2 Appendix). Like Hyp femurs, NPT2aKO femurs exhibit a significant decrease in trabecular bone volume fraction (BV/TV) compared to WT. NPT2aKO femurs also have a decrease in trabecular number (Tb.N) and increase in trabecular spacing (Tb.Sp) relative to WT, although these parameters are significantly improved compared to Hyp mice. NPT2aKO femurs have normal trabecular thickness (Tb.Th) (S2 Appendix).

### VDR^f/f;DMP1Cre+^ or NPT2aKO tibiae and calvariae exhibit enlarged osteocyte lacunar area and impaired canalicular organization

**Fig 4:**
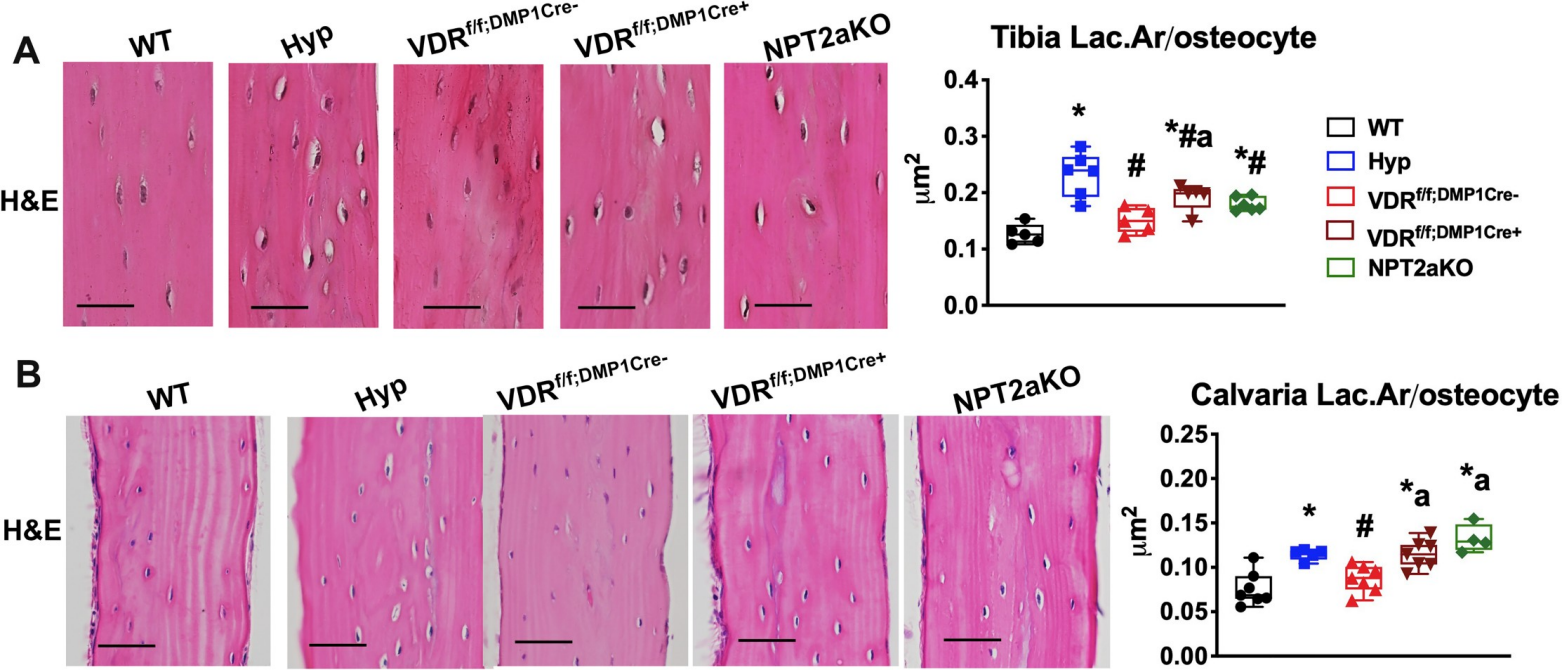
The tibial cortices and calvariae of VDR^f/f;DMP1Cre+^ and NPT2aKO mice have enlarged osteocyte lacunae. H&E stain of WT, Hyp, VDR^f/f;DMP1Cre-^, VDR^f/f;DMP1Cre+^ and NPT2aKO tibial cortices (d30) (A) and calvaria (d75) (B). Histomorphometric quantitation of osteocyte number and lacunar area normalized to the number of osteocytes (Lac.Ar/osteocyte) were performed proximal and contralateral to the tibiofibular junction and in the bilateral parietal bones. Data are representative of that obtained from at least 5 mice per genotype, with each group including 2–4 mice of either sex. Scale bar = 20 μm in (A). Scale bar = 40 μm in (B). * p<0.05 vs WT, # p<0.05 vs Hyp, a p<0.05 vs VDR^f/f;DMP1Cre-^.

Analysis of the osteocyte LCN phenotype in VDR^f/f;DMP1Cre+^ mice will address the hypothesis that 1,25D acts directly on osteocytes to regulate lacunar morphology and canalicular structure. Also, characterizing the LCN phenotype in NPT2aKO mice will determine if systemic hypophosphatemia in the setting of high 1,25D alters osteocyte lacunar and canalicular organization. To evaluate the osteocyte lacunar area, tibial cortices and calvariae of Hyp, VDR^f/f;DMP1Cre+^, and NPT2aKO mice and corresponding WT and VDR^f/f;DMP1Cre-^ controls were analyzed. Hematoxylin and eosin (H&E) stain demonstrates that the lacunar area of Hyp, VDR^f/f;DMP1Cre+^, and NPT2aKO tibial cortices (Fig 4A) and calvariae (Fig 4B) are larger than that of control. Histomorphometric analyses were performed to quantify the lacunar area/osteocyte (Lac.Ar/osteocyte) (Fig 4A and 4B). Consistent with our previous analyses [15], Hyp tibiae and calvariae have larger Lac.Ar/osteocyte than WT. The Lac.Ar/osteocyte in VDR^f/f;DMP1Cre+^ and NPT2aKO tibial cortices were found to be larger than WT, but still smaller than that of Hyp tibiae (Fig 4A). The Lac.Ar/osteocyte in both VDR^f/f;DMP1Cre+^ and NPT2aKO calvariae was indistinguishable from that in Hyp calvariae and larger than WT calvariae (Fig 4B). Lac.Ar/osteocyte in VDR^f/f;DMP1Cre-^ tibiae and calvariae are normal. These results suggest impaired 1,25D action in osteocytes and hypophosphatemia despite increased 1,25D levels lead to enhanced osteocyte-mediated perilacunar matrix resorption.

**Fig 5:**
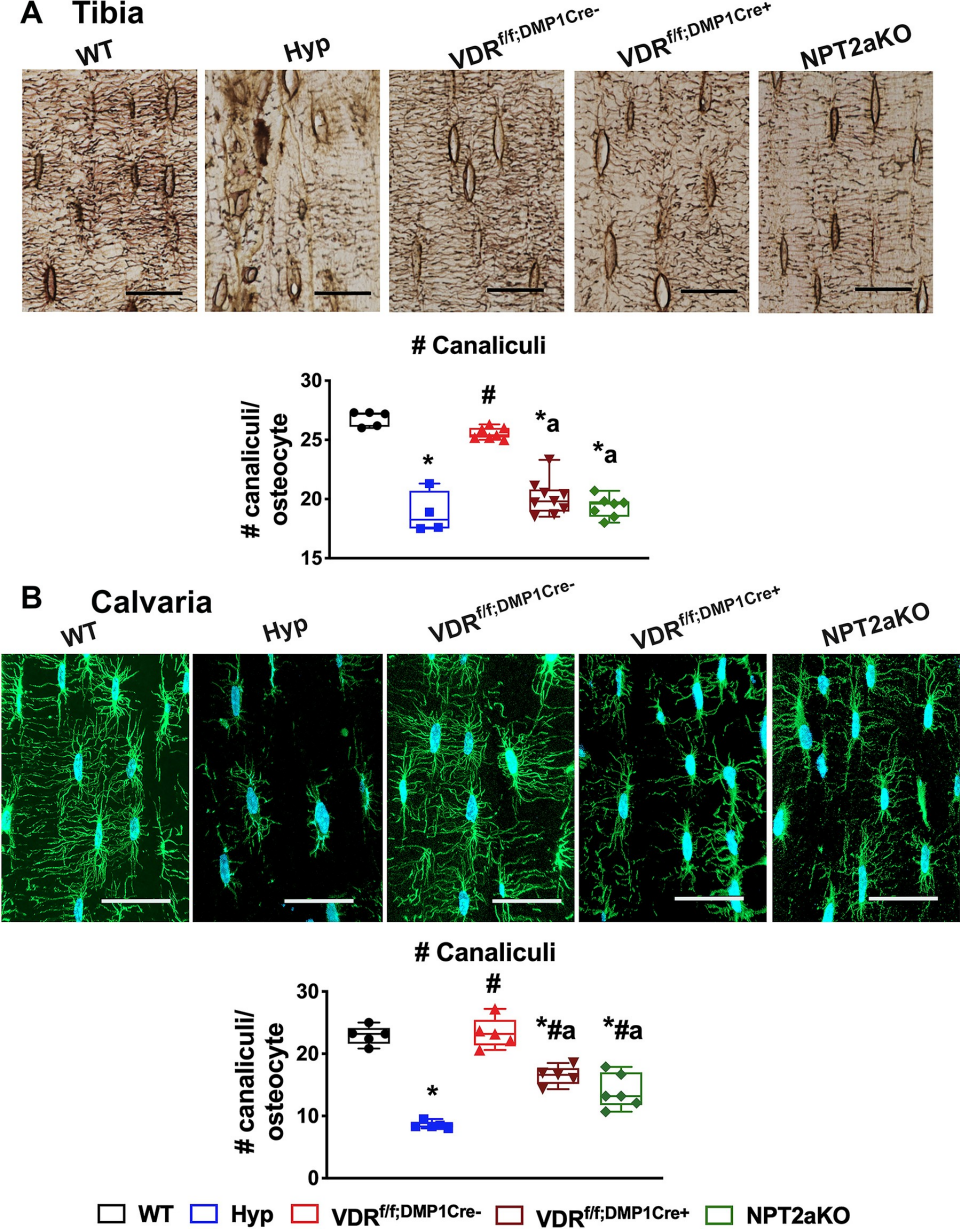
The tibial cortices of VDR^f/f;DMP1Cre+^ and NPT2aKO have impaired canalicular organization. (A) Silver stain of the canalicular network in d75 WT, Hyp, VDR^f/f;DMP1Cre-^, VDR^f/f;DMP1Cre+^ and NPT2aKO tibial cortices. Quantitation of number of canaliculi/osteocyte. Data are representative of that obtained from at least 5 mice per genotype, with WT and Hyp mice including 2–3 mice of either sex and all other groups including 3–5 mice of either sex. (B) Phalloidin stain of the canalicular network in d30 calvariae from WT, Hyp, VDR^f/f;DMP1Cre-^, VDR^f/f;DMP1Cre+^ and NPT2aKO mice. Number of canaliculi per osteocyte are quantified. Data are representative of that obtained from at least 5 mice per genotype, with each group including 2–3 mice of either sex. Scale bars = 20 μm in (A) and (B). * p<0.05 vs WT, # p<0.05 vs Hyp, a p<0.05 vs VDR^f/f;DMP1Cre-^, b p<0.05 vs VDR^f/f;DMP1Cre+^.

The abnormal canalicular organization observed in Hyp mice is significantly improved by treatment with 1,25D or FGF23Ab, suggesting that impaired 1,25D action or hypophosphatemia contributes to the canalicular abnormalities seen in Hyp bones. Silver stain of WT and VDR^f/f;DMP1Cre-^ tibiae (Fig 5A) and phalloidin stain of WT and VDR^f/f;DMP1Cre-^ calvariae (Fig 5B) confirm that they have dense and abundant canaliculi, forming an extensive network of dendrites that extend around the circumference of each osteocyte. In contrast to these controls, Hyp tibiae and calvariae have sparse and short dendrites. Consistent with alterations in canalicular organization seen in Hyp mice, both VDR^f/f;DMP1Cre+^ and NPT2aKO tibiae and calvariae also have decreased number of canaliculi extending from the osteocytes (Fig 5A and 5B). These data support the hypothesis that osteocyte-specific 1,25D action and hypophosphatemia regulate osteocyte canalicular organization.

### Osteocyte enriched bone from VDR^f/f;DMP1Cre+^ and NPT2aKO mice have enhanced expression of markers of osteocyte-mediated perilacunar remodeling

**Fig 6:**
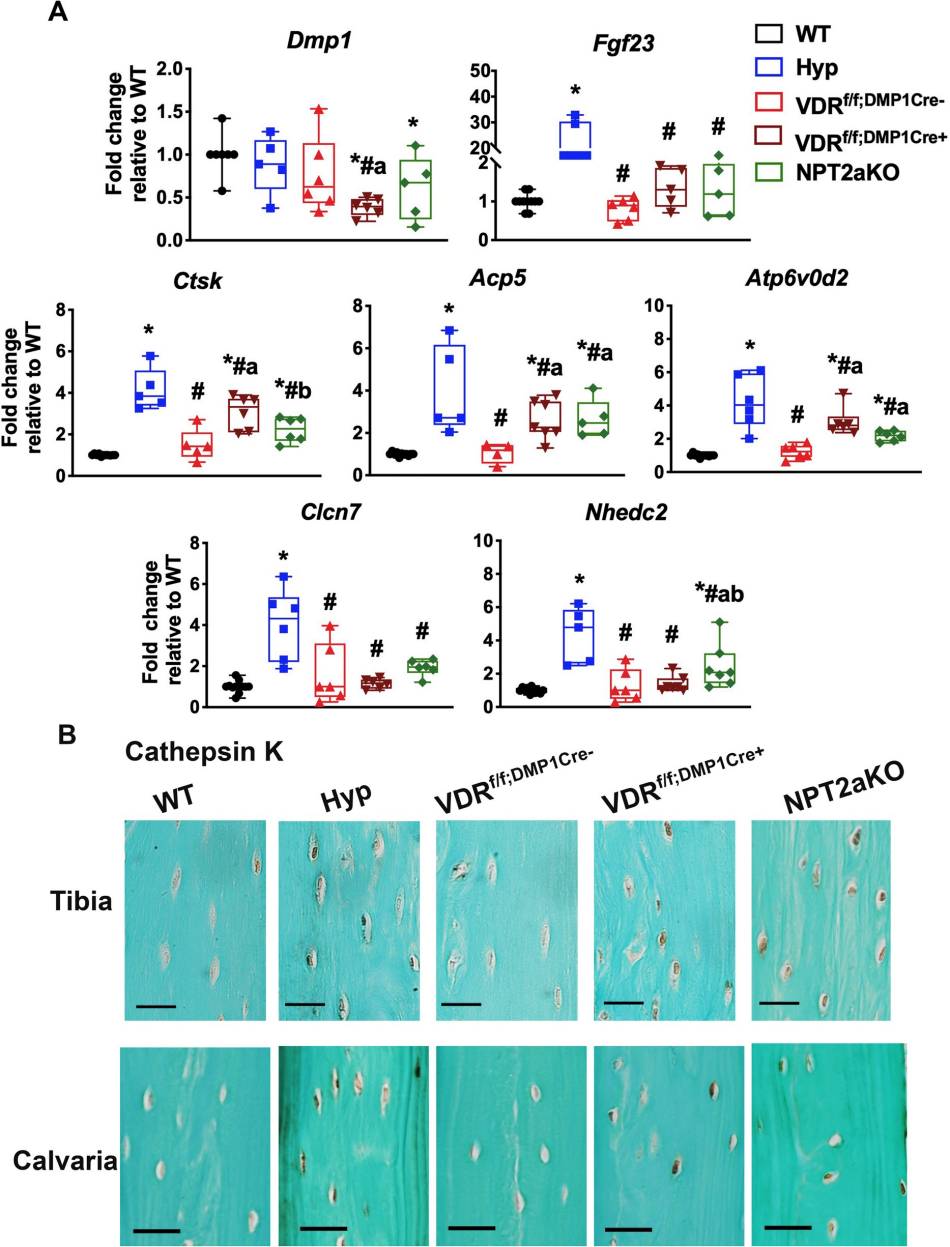
VDR^f/f;DMP1Cre+^ and NPT2aKO osteocytes have increased expression of genes that regulate osteocyte perilacunar remodeling. (A) RNA was isolated from osteocyte-enriched tibiae from d30 WT, Hyp, VDR^f/f;DMP1Cre-^, VDR^f/f;DMP1Cre+^ and NPT2aKO mice and subjected to RT-qPCR to quantitate mRNA expression for *Dmp1*, *Fgf23*, and markers of perilacunar remodeling. Data normalized to actin and represented as fold change relative to WT. Data are representative of that obtained from at least 5 mice per genotype, with WT mice including 5–6 mice of either sex, Hyp mice including 2–3 mice of either sex, and all other groups including 3–4 mice of either sex. * p<0.05 vs WT, # p<0.05 vs Hyp, a p<0.05 vs VDR^f/f;DMP1Cre-^, b p<0.05 vs VDR^f/f;DMP1Cre+^. (B) Immunohistochemistry for cathepsin K in d30 WT, Hyp, VDR^f/f;DMP1Cre-^, VDR^f/f;DMP1Cre+^ and NPT2aKO tibial cortices and calvaria. Scale bars = 20 μm. Data are representative of that obtained from at least 5 mice per genotype, with each group including 2–3 mice of either sex.

Osteocyte expression of genes used by osteoclasts to resorb bone is enhanced during increased perilacunar matrix resorption [6]. Our previous studies demonstrated that the enlarged osteocyte lacunae in Hyp tibiae are associated with enhanced osteocyte expression of these perilacunar matrix resorption genes, and expression of these genes is normalized with 1,25D treatment [15]. Because the osteocyte lacunae in VDR^f/f;DMP1Cre+^ and NPT2aKO mice are also enlarged, osteocyte enriched tibial RNA was isolated to determine if osteocytes from these mice had enhanced expression of genes that regulate perilacunar remodeling. VDR^f/f;DMP1Cre+^ tibial osteocytes have decreased *Dmp1* expression relative to WT and VDR^f/f;DMP1Cre-^ controls. All other groups of mice have normal *Dmp1* expression (Fig 6A). Consistent with the Hyp phenotype, Hyp tibial osteocytes have enhanced expression of *Fgf23* as well as bone resorption genes cathepsin K (*Ctsk*) and tartrate-resistant acid phosphatase (*Acp5*) and genes that regulate the acidification of the extracellular environment during matrix resorption, including ATPase H+ transporting lysosomal V0 subunit (*Atp6v0d2*), chloride channel 7 (*Clcn7*), and Na+/H+ exchanger domain containing 2 (*Nhedc2*) (Fig 6A). Similarly, VDR^f/f;DMP1Cre+^ and NPT2aKO osteocytes have increased mRNA expression of *Ctsk*, *A*c*p5*, and *Atp6v0d2* relative to WT and VDR^f/f;DMP1Cre-^ controls, though mRNA expression of these genes are decreased relative to Hyp osteocytes. Neither VDR^f/f;DMP1Cre+^ nor NPT2aKO tibial osteocytes have altered mRNA expression of *Clcn7*. Expression of *Nhedc2* is not different in VDR^f/f;DMP1Cre+^ tibiae relative to controls; however, it is increased in NPT2aKO tibiae to a level similar to that observed in Hyp tibiae (Fig 6A). Immunohistochemical analyses confirmed that osteocytes from Hyp, VDR^f/f;DMP1Cre+^, and NPT2aKO tibiae and calvariae overexpress Ctsk (Fig 6B).

### 1,25D and phosphate suppress the expression of genes that regulate perilacunar remodeling in osteocytes *in vitro*

**Fig 7:**
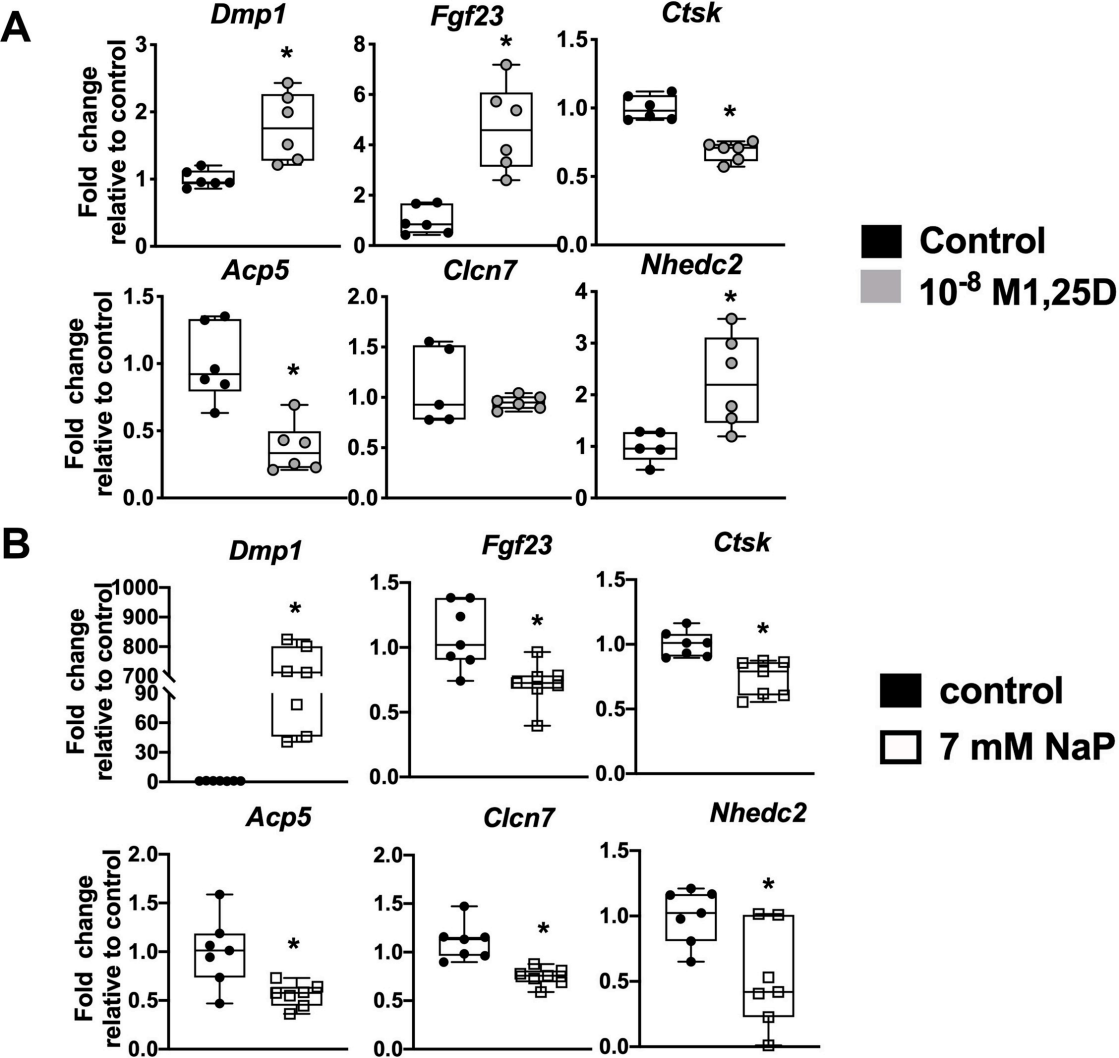
1,25D and phosphate treatment of Ocy454 cells decreases expression of genes implicated in perilacunar matrix resorption. Ocy454 cells were treated with 24 hours of control or 10^-8^M 1,25D (A) or 7 mM sodium sulfate (control) or sodium phosphate for 8 hours (B). Gene expression of *Dmp1*, *Fgf23*, and markers of perilacunar remodeling were quantitated. Data are representative of that obtained from at least 5 biological replicates. * p<0.05 vs control.

VDR^f/f;DMP1Cre+^ and NPT2aKO mice have enlarged osteocyte lacunae accompanied by enhanced osteocytic expression of genes which regulate matrix resorption. To determine the direct effects of 1,25D and phosphate on expression of these genes *in vitro*, Ocy454 osteocytic cells were treated with 1,25D or phosphate. Since VDR^f/f;DMP1Cre+^ osteocytes have decreased *Dmp1* expression and increased expression of select matrix resorption genes like *Ctsk* and *Acp5* (Fig 6A), it is hypothesized that treatment of the Ocy454 cells with 1,25D will exert the opposite effects on the expression of those genes. Treatment of Ocy454 cells with 10^-8^M 1,25D increased expression of *Dmp1*, *Fgf23*, and *Nhedc2*, decreased expression of markers of matrix resorption *Ctsk* and *Acp5*, and did not alter the expression of *Clcn7* (Fig 7A). Likewise, osteocytes from NPT2aKO mice also have enhanced expression of perilacunar matrix remodeling genes (Fig 6A), therefore it is expected that treatment of Ocy454 cells with phosphate will decrease the expression of those genes. Treatment of Ocy454 cells with 7 mM sodium phosphate led to increased expression of *Dmp1* and decreased expression of *Fgf23*, as well as decreased expression of perilacunar remodeling markers *Ctsk*, *Acp5*, *Clcn7*, and *Nhdec2* (Fig 7B). Expression of *Atp6v0d2* was not detected in all samples, and therefore inconsistent. These results support the hypothesis that 1,25D and phosphate act independently on osteocytes to modulate resorption of the surrounding mineral matrix.

## Discussion

XLH is characterized by elevated serum levels of FGF23 which leads to impaired production of 1,25D and hypophosphatemia [26, 27]. Previous data showed that Hyp mice have dramatically abnormal cortical and trabecular skeletal microarchitecture, which was improved with either 1,25D or FGF23Ab treatment [15]. These data suggest restoration of 1,25D and/or phosphate levels contribute to the improved microarchitecture observed in Hyp mice. Mice with global ablation of the Vdr [28] or the vitamin D 1-alpha-hydroxylase [29] maintained on a regular diet have significantly decreased cortical thickness and trabecular bone volume fraction in the setting of hypocalcemia, hypophosphatemia, and secondary hyperparathyroidism. Consistent with results reported by Lieben et al. [30], our data demonstrates that VDR^f/f;DMP1Cre+^ mice have normal cortical and trabecular microarchitecture, implying that the alterations in microarchitecture observed in Hyp mice or mice null for 1,25D action fed a regular diet are due to lack of 1,25D action in cells other than osteocytes. Previous studies reported that overexpression of the *Vdr* in osteocalcin expressing cells leads to increased bone mass, thus demonstrating a role for vitamin D action in regulating bone formation by mature osteoblasts [31].

In contrast, NPT2aKO mice have a mild decrease in cortical thickness and tissue mineral density and significantly impaired trabecular organization, despite having high serum 1,25D levels. Both Hyp mice [18] and mice fed a low phosphate diet [32] have decreased bone volume fraction. In particular, Hyp bones have a dramatic decrease in trabeculae with a significant decrease in trabecular number and increase in trabecular spacing [18]. The similar trabecular phenotype of NPT2aKO mice to these mouse models points to an important role for phosphate in maintaining normal trabecular structure.

Hyp mice have enlarged periosteocytic lacunae and sparse canaliculae. This abnormal lacuno-canalicular (LCN) organization is restored with 1,25D or FGF23Ab therapy, suggesting that 1,25D and/or phosphate play roles in regulating LCN remodeling [15]. In support of the hypothesis that 1,25D action is important for maintaining LCN morphology, cortical bone from humans deficient in vitamin D have higher osteocyte lacunar volume [33] and poor canalicular structure [34] compared to vitamin D sufficient controls and mice null for Vdr fed a normal diet have larger lacunar area than WT. Since bones from these human and murine models of vitamin D deficiency are characterized by osteomalacia, it is not clear if the LCN abnormalities are due to decreased 1,25D signaling or impaired mineralization. Because VDR^f/f;DMP1Cre+^ mice have normal cortical microarchitecture as well as enlarged osteocyte lacunae and defective canalicular organization, this data demonstrates that 1,25D acts directly on osteocytes to modulate LCN organization and suggest that the abnormalities in the LCN phenotype seen in VDR^f/f;DMP1Cre+^ mice is not a result of altered skeletal mineralization.

PTH/PTHrP acts to resorb the mineral matrix surrounding osteocytes [6, 8]. Since either 1,25D or FGF23Ab treatment of Hyp mice normalizes serum PTH levels [18] and improves the LCN phenotype [15], Hyp mice may have enlarged lacunae in part due to increased serum PTH levels. Since VDR^f/f;DMP1Cre+^ mice have normal serum PTH levels and NPT2aKO mice have decreased serum PTH levels, the enlarged lacunae in these mice are not a result of increased PTH action.

Furthermore, the abnormal LCN phenotype observed in NPT2aKO mice indicate that the high 1,25D levels found in these mice are not able to compensate for the enlarged osteocyte lacunae and impaired canalicular organization seen in the setting of hypophosphatemia. These findings identify a novel role for phosphate in regulating periosteocytic lacunar remodeling and canalicular structure. The enlarged lacunae in NPT2aKO tibiae is accompanied by increased expression of osteocyte perilacunar matrix resorption genes, suggesting low serum phosphate levels can be sensed by osteocytes and stimulate perilacunar matrix resorption. Feeding Vdr null mice a high calcium high phosphate diet normalizes osteocyte lacunar size, further supporting that hypophosphatemia impairs osteocyte lacunar morphology [34]. The Vdr null mice fed a regular diet, which have secondary hyperparathyroidism, may have enlarged lacunae in part be due to enhanced PTH action. In contrast, NPT2aKO mice have decreased serum PTH levels, thus supporting a role for conditions other than hyperparathyroidism, such as hypophosphatemia, in enhancing osteocyte mediated mineral matrix resorption.

Although 1,25D treatment of Hyp mice normalizes osteocyte lacunar size and expression of bone resorption genes [15], the elevated 1,25D levels in NPTaKO mice are not able to prevent the enlargement of the osteocyte lacunae and the increase in osteocyte expression of genes classically used by osteoclasts to resorb bone observed in these mice. This may be because 1,25D therapy not only compensates for the decreased 1,25D action, but also significantly increases serum phosphate in Hyp mice [18]. However, the elevated 1,25D levels in NPT2aKO mice, which result from the physiologic response to the low serum phosphate, are not able to improve serum phosphate because 1,25D’s actions are largely due to stabilizing renal brush border membrane NPT2a which is absent in the NPT2a knockout mice [35]. This suggests that improvement in the LCN phenotype in Hyp mice treated with 1,25D was not only due to increased 1,25D action, but also the increase in serum phosphate, thus further underscoring the importance of phosphate in regulating osteocyte perilacunar matrix resorption.

The abnormal LCN phenotype observed in Hyp, VDR^f/f;DMP1Cre+^ and NPT2aKO mice suggest there may be defects in osteocyte maturation and/or function in these mice. The alterations in LCN phenotype are less severe in the VDR^f/f;DMP1Cre+^ and NPT2aKO mice than Hyp mice. One possibility for the more dramatic phenotype seen in Hyp mice is that the low 1,25D and phosphate levels are additive in contributing to the LCN abnormalities observed in XLH. In addition, Hyp mice have dramatically impaired cortical microarchitecture, while VDR^f/f;DMP1Cre+^ and NPT2aKO do not have significant alterations in cortical microarchitecture. This suggests impaired skeletal mineralization, independent of the roles of phosphate and 1,25D in acting directly on osteocytes to regulate LCN remodeling, also contributes to the modulation of perilacunar matrix resorption and canalicular organization. Further studies would be necessary to dissect the specific role of mineralization on osteocyte LCN remodeling.

The significantly abnormal LCN phenotype in Hyp long bones is associated with dramatically impaired cortical microarchitecture and inferred biomechanical parameters (Fig 3) and decreased bone strength on torsional biomechanical testing [18]. While it is possible that the severe osteocyte LCN abnormalities seen in Hyp mice lead to decreased bone strength, the impaired microarchitecture and intrinsic defects in osteocytes and osteoblasts due to the PHEX mutation [36, 37] likely also contribute to the impaired inferred biomechanical parameters and strength observed in the Hyp bones. In contrast, VDR^f/f;DMP1Cre+^ and NPT2aKO bones have an intermediate LCN phenotype that is less severe than Hyp. VDR^f/f;DMP1Cre+^ mice have normal cortical microarchitecture and NPT2aKO mice have mildly decreased cortical thickness and tissue mineral density, suggesting the LCN abnormalities in VDR^f/f;DMP1Cre+^ and NPT2aKO mice may not be severe enough to impair the inferred biomechanical parameters. Further studies are needed to elucidate whether enlarged osteocyte lacunar and abnormal canalicular structure directly contribute to impaired bone strength.

Similar to lactating mice [6], the enlarged periosteocytic lacunae in Hyp mice are accompanied by increased osteocyte expression of genes used by osteoclasts to resorb mineralized matrix [15]. Both 1,25D and FGF23Ab treatment improved the osteocyte expression of bone resorption genes in Hyp mice [15], pointing to roles for 1,25D and phosphate in modulating periosteocytic lacunar remodeling. The larger size of the osteocytic lacunae in both VDR^f/f;DMP1Cre+^ and NPT2aKO mice correlates with increased expression of lacunar remodeling genes and treatment of Ocy454 cells with either 1,25D or phosphate suppresses expression of these genes. These data demonstrate that 1,25D and phosphate are each independently able to act directly on osteocytes to inhibit periosteocytic lacunar matrix resorption. In support of our findings that 1,25D can suppress mineral matrix release, previous studies have shown that treatment of rodents with active vitamin D compounds like eldecalcitol [38], alfacalcidol [39], and calcitriol [40] inhibit bone resorption. Furthermore, incubation of phosphate-treated primary osteoclasts with bone resulted in a decrease in the number and size of bone resorption pits [41]. Although the expression of bone resorption genes was not reported, these studies demonstrate that phosphate inhibits osteoclastic bone resorptive activity [41]. Since osteocytes can function like osteoclasts in that they express bone resorption genes and remodel their surrounding matrix [6], these studies support our findings that osteocyte mediated mineral matrix resorption is similarly suppressed by phosphate. Because PTH/PTHrP acts to resorb the mineral surrounding osteocytes to meet the high calcium demand in physiologic states like lactation [6], it is possible that the low phosphate state can be sensed by osteocytes and similarly enhance osteocyte lacunar matrix resorption to contribute to the restoration of normophosphatemia.

Previous *in vitro* studies suggested that osteocytes are able to sense extracellular phosphate concentration and that their response to phosphate is dependent on the concentration of phosphate [42]. Treatment of IDG-SW3 osteocytes with a lower concentration of phosphate (4 mM) induced expression of *Fgf23*, while treatment of the cells with higher concentration of phosphate (10 mM) suppressed *Fgf23* expression [42]. Our results are consistent with these studies, as treatment of Ocy454 cells with a higher concentration of phosphate (7mM) similarly decreased *Fgf23* expression. Although it has not been reported that *Nhdec2* expression is a target of 1,25D action, our data demonstrates that 1,25D increases *Nhdec2* expression in Ocy454 cells. In contrast, hypophosphatemia enhances *Nhdec2* expression in NPT2aKO osteocytes and phosphate treatment of Ocy454 cells decreases expression of this mineral resorption gene. These findings suggest that 1,25D and phosphate may have different target genes, and therefore differentially regulate select osteocyte perilacunar remodeling genes.

In contrast to our results, RNASeq analyses demonstrated that treatment of IDG-SW3 osteocytes with 1,25D did not change the expression of perilacunar matrix resorption genes [43]. The IDG-SW3 cells were differentiated for 35 days, as opposed to the Ocy454 cells in our studies which were differentiated for one week. The characteristics of the two cell lines are different in that at least 2–3 weeks of differentiation are required for increased expression of mature osteocyte-specific genes like *Fgf23* in IDG-SW3 cells [44], but increased expression of osteocyte-specific genes in Ocy454 cells is observed starting at one week of differentiation [24]. Since the IDG-SW3 cells were differentiated for a few weeks beyond the start of the increased expression of osteocyte genes, it is possible that the Ocy454 cells in our studies are less differentiated relative to the IDG-SW3 cells used in the previous RNASeq studies. The differences in osteocyte differentiation as well as experimental conditions and the differences between the two *in vitro* cell lines could account for the dissimilarity in results.

Previous studies demonstrated that 1,25D enhances FGF23 expression by inducing the FGF23 promoter [45]. Consistent with this, treatment of Ocy454 cells with 1,25D increased FGF23 expression. Similarly, it was previously shown that treatment of Hyp mice with 1,25D also dramatically increases FGF23 expression in bone and circulating FGF23 levels [18]. Despite this increase in FGF23 expression, 1,25D treatment of Hyp mice paradoxically increases serum phosphate levels and decreases urinary phosphate wasting by increasing the expression of NPT2a on the renal brush border membrane [18, 46]. Administering phosphate therapy to individuals affected by XLH increases phosphate wasting and leads to secondary hyperparathyroidism and nephrocalcinosis, where the dose of phosphate correlates with the severity of nephrocalcinosis; low doses of 1,25D are given in combination with phosphate to XLH patients to suppress serum PTH levels [47, 48]. Therefore, the beneficial effects of 1,25D on phosphate homeostasis as well as the LCN and skeletal phenotype in Hyp mice [15, 18] supports optimizing 1,25D action in patients with XLH.

In conclusion, these studies demonstrate that 1,25D acts directly on osteocytes to regulate perilacunar remodeling and canalicular organization. They also demonstrate that the high 1,25D levels in the NPT2aKO mice cannot compensate for the effects of hypophosphatemia on LCN remodeling, thus showing that the low phosphate state stimulates osteocyte lacunar matrix resorption to potentially help maintain phosphate homeostasis and impairs canalicular structure. Consistent with our hypothesis that 1,25D and phosphate directly regulates perilacunar matrix resorption, 1,25D and phosphate treatment of *in vitro* osteocytes was each able to decrease the expression of bone resorption genes. Since XLH is characterized by low serum 1,25D levels and hypophosphatemia accompanied by an abnormal LCN phenotype [15], these findings emphasize the importance of restoring 1,25D and phosphate action in patients with XLH.

## Supporting information

S1 Appendix Representative microCT images of distal femurs. (TIF)

S2 Appendix Femoral trabecular microarchitecture parameters in Hyp, VDR^f/f;DMP1Cre+^, NPT2aKO, and control mice. MicroCT was performed on d30 femurs isolated from WT, Hyp, VDR^f/f;DMP1Cre-^, VDR^f/f;DMP1Cre+^ and NPT2aKO mice. (A) Representative trabecular images of microCT scans are shown. Scale bar = 0.5 mm. (B) Trabecular microCT parameters (bone volume fraction (BV/TV), trabecular number (Tb.N), trabecular spacing (Tb.Sp), and trabecular thickness (Tb.Th). Data are representative of that obtained from at least 5 mice per genotype, with NPT2aKO mice including 2–3 mice of either sex and all other groups including 3–4 mice of either sex. * p<0.05 vs WT, # p<0.05 vs Hyp, a p<0.05 vs VDR^f/f;DMP1Cre-^, b p<0.05 vs VDR^f/f;DMP1Cre+^.
(TIF)
