# Supplementary figures and images for: Impaired 1,25 dihydroxyvitamin D3 action and hypophosphatemia underlie the altered lacuno-canalicular remodeling observed in the Hyp mouse model of XLH
Source: PLoS One. 2021 May 27;16(5):e0252348. doi: 10.1371/journal.pone.0252348 (PMC8158930; doi:10.1371/journal.pone.0252348)

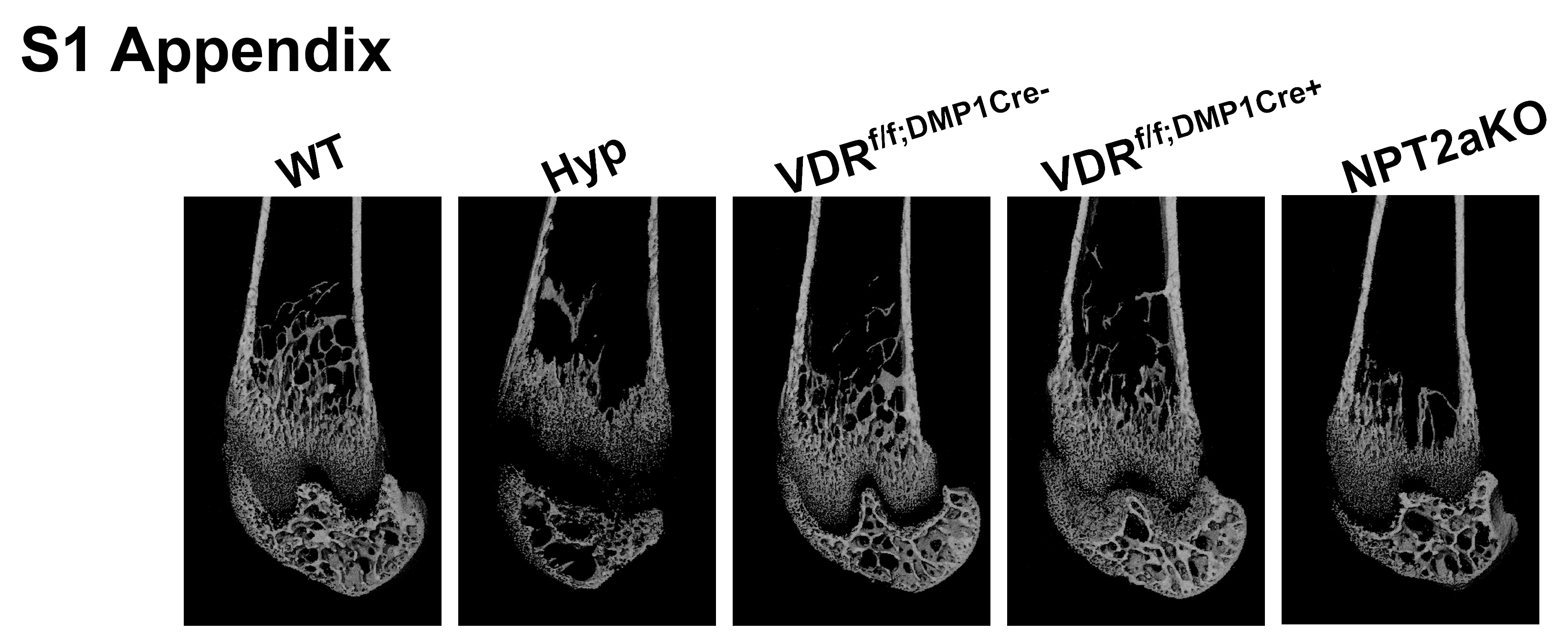

Supplement: S1 Appendix — (TIF) [file pone.0252348.s001.tif]

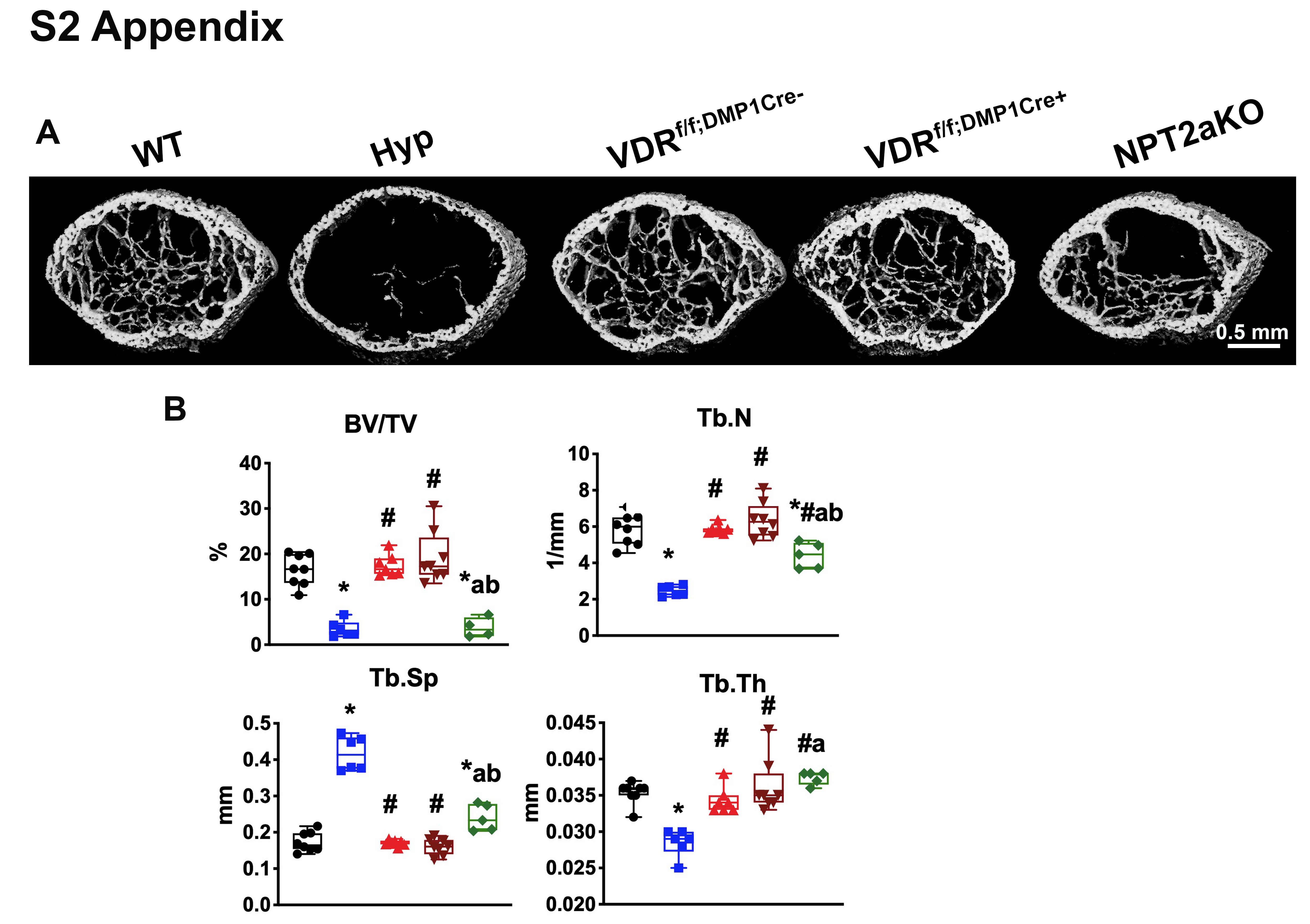

Supplement: S2 Appendix — MicroCT was performed on d30 femurs isolated from WT, Hyp, VDRf/f;DMP1Cre-, VDRf/f;DMP1Cre+ and NPT2aKO mice. (A) Representative trabecular images of microCT scans are shown. Scale bar = 0.5 mm. (B) Trabecular microCT parameters (bone volume fraction (BV/TV), trabecular number (Tb.N), trabecular spacing (Tb.Sp), and trabecular thickness (Tb.Th). Data are representative of that obtained from at least 5 mice per genotype, with NPT2aKO mice including 2–3 mice of either sex and all other groups including 3–4 mice of either sex. * p<0.05 vs WT, # p<0.05 vs Hyp, a p<0.05 vs VDRf/f;DMP1Cre-, b p<0.05 vs VDRf/f;DMP1Cre+. (TIF) [file pone.0252348.s002.tif]
